# Frequent k- *ras* -2 mutations and *p16*^INK4A^methylation in hepatocellular carcinomas in workers exposed to vinyl chloride

**DOI:** 10.1054/bjoc.2000.1675

**Published:** 2001-04

**Authors:** M Weihrauch, M Benicke, G Lehnert, C Wittekind, R Wrbitzky, A Tannapfel

**Affiliations:** Institute of Occupational and Environmental Medicine, University of Hannover; Institute of Pathology, University of Leipzig; Institute of Occupational, Social and Environmental Medicine, University of Erlangen-Nuremberg, Germany

**Keywords:** vinyl chloride, HCC, *ras* mutation, *p16* methylation

## Abstract

Vinyl chloride (VC) is a know animal and human carcinogen associated with liver angiosarcomas (LAS) and hepatocellular carcinomas (HCC). In VC-associated LAS mutations of the K- *ras* -2 gene have been reported; however, no data about the prevalence of such mutations in VC associated HCCs are available. Recent data indicate K- *ras* -2 mutations induce *P16* methylation accompanied by inactivation of the *p16* gene. The presence of K- *ras* -2 mutations was analysed in tissue from 18 patients with VC associated HCCs. As a control group, 20 patients with hepatocellular carcinoma due to hepatitis B (*n* = 7), hepatitis C (*n* = 5) and alcoholic liver cirrhosis (*n* = 8) was used. The specific mutations were determined by direct sequencing of codon 12 and 13 of the K- *ras* -2 gene in carcinomatous and adjacent non-neoplastic liver tissue after microdissection. The status of *p16* was evaluated by methylation-specific PCR (MSP), microsatellite analysis, DNA sequencing and immunohistochemical staining. All patients had a documented chronic quantitated exposure to VC (average 8883 ppmy, average duration: 245 months). K- *ras* -2 mutations were found in 6 of 18 (33%) examined VC-associated HCCs and in 3 cases of adjacent non-neoplastic liver tissue. There were 3 G → A point mutations in the tumour tissue. All 3 mutations found in non-neoplastic liver from VC-exposed patients were also G → A point mutations (codon 12- and codon 13-aspartate mutations). Hypermethylation of the 5′ CpG island of the *p16* gene was found in 13 of 18 examined carcinomas (72%). Of 6 cancers with K- *ras* -2 mutations, 5 specimens also showed methylated *p16*. Within the control group, K- *ras* -2 mutation were found in 3 of 20 (15%) examined HCC. *p16* methylation occurred in 11 out of 20 (55%) patients. K- *ras* -2 mutations and *p16* methylation are frequent events in VC associated HCCs. We observed a K- *ras* -2 mutation pattern characteristic of chloroethylene oxide, a carcinogenic metabolite of VC. Our results strongly suggest that K- *ras* -2 mutations play an important role in the pathogenesis of VC-associated HCC. © 2001 Cancer Research Campaignhttp://www.bjcancer.com


*British Journal of Cancer* (2001) **84**(7), 982–989
© 2001 Cancer Research Campaign
doi: 10.1054/ bjoc.2000.1675, available online at http://www.idealibrary.com on IDEAL®

http://www.bjcancer.com

### Frequent k-*ras*-2 mutations and *p16*$^{INK4A}$ methylation in hepatocellular carcinomas in workers exposed to vinyl chloride

**M Weihrauch[1], M Benicke[2], G Lehnert[3], C Wittekind[2], R Wrbitzky[1] and A Tannapfel[2]**

[1]Institute of Occupational and Environmental Medicine, University of Hannover; [2] Institute of Pathology, University of Leipzig; [3]Institute of Occupational, Social and Environmental Medicine, University of Erlangen-Nuremberg, Germany

**Summary** Vinyl chloride (VC) is a know animal and human carcinogen associated with liver angiosarcomas (LAS) and hepatocellular carcinomas (HCC). In VC-associated LAS mutations of the K-*ras*-2 gene have been reported; however, no data about the prevalence of such mutations in VC associated HCCs are available. Recent data indicate K-*ras*-2 mutations induce *P16* methylation accompanied by inactivation of the *p16* gene. The presence of K-*ras*-2 mutations was analysed in tissue from 18 patients with VC associated HCCs. As a control group, 20 patients with hepatocellular carcinoma due to hepatitis B ($n = 7$), hepatitis C ($n = 5$) and alcoholic liver cirrhosis ($n = 8$) was used. The specific mutations were determined by direct sequencing of codon 12 and 13 of the K-*ras*-2 gene in carcinomatous and adjacent non-neoplastic liver tissue after microdissection. The status of *p16* was evaluated by methylation-specific PCR (MSP), microsatellite analysis, DNA sequencing and immunohistochemical staining. All patients had a documented chronic quantitated exposure to VC (average 8883 ppmy, average duration: 245 months). K-*ras*-2 mutations were found in 6 of 18 (33%) examined VC-associated HCCs and in 3 cases of adjacent non-neoplastic liver tissue. There were 3 G $\rightarrow$ A point mutations in the tumour tissue. All 3 mutations found in non-neoplastic liver from VC-exposed patients were also G $\rightarrow$ A point mutations (codon 12- and codon 13-aspartate mutations). Hypermethylation of the 5′ CpG island of the *p16* gene was found in 13 of 18 examined carcinomas (72%). Of 6 cancers with K-*ras*-2 mutations, 5 specimens also showed methylated *p16*. Within the control group, K-*ras*-2 mutation were found in 3 of 20 (15%) examined HCC. *p16* methylation occurred in 11 out of 20 (55%) patients. K-*ras*-2 mutations and *p16* methylation are frequent events in VC associated HCCs. We observed a K-*ras*-2 mutation pattern characteristic of chloroethylene oxide, a carcinogenic metabolite of VC. Our results strongly suggest that K-*ras*-2 mutations play an important role in the pathogenesis of VC-associated HCC. © 2001 Cancer Research Campaign http://www.bjcancer.com

**Keywords**: vinyl chloride; HCC; *ras* mutation; *p16* methylation

Vinyl chloride (VC) is a colourless toxic gas used extensively in the plastic industry as a refridgerant and an intermediate in organic synthesis. VC is rapidly absorbed following respiratory exposure and is primarily metabolized in the liver via the microsomal cytochrome P450 monooxygenase system to the electrophilic metabolites: chloroethylene oxide (CEO) and chloroacetaldehyde (CAA) (el Ghiassassi et al, 1998). CEO and CAA react with DNA bases to form mutagenic adducts in bacterial and mammalian cells (Thompson et al, 1985; Barbin, 1999). In humans, a causal relationship has been demonstrated between occupational exposure to VC and liver angiosarcomas (LAS) (Tamburro et al, 1984; Hollstein et al, 1994). In 1983 Evans et al reported 2 cases of hepatocellular carcinomas (HCC) among VC workers (Evans et al, 1983). Several epidemiological studies again reported this association in humans (Trivers et al, 1995; Marion et al, 1996). Increased morbidity odds ratios of HCC among VC workers were recently described (Du and Wang, 1998). However, in cohort studies, primary HCC and LAS are not always delineated, and the status of the adjacent non-neoplastic liver tissue is not available in most reported data (Heldaas et al, 1984). Exact data concerning the amount of VC exposure have not been presented within the studies published. Current literature demonstrates mutation analysis of the proto-oncogene *ras* has been analysed in LAS due to VC (DeVivo et al, 1994; Luo et al, 1998) and in animal models (Soman and Wogan, 1993; Froment et al, 1994; Barbin et al, 1997; Boivin-Angele et al, 2000); yet, no published data are available for human HCCs associated with VC exposure. After *ras*, one of the earliest and most potent oncogenes identified in human cancer, the tumour suppressor gene *p16* is one of the most frequently altered genes observed in various malignancies (Xing et al, 1999). The *p16* gene product encodes a negative regulatory protein preventing the cell cycle progression from $G_1$ to S-phase (Roussel, 1999). Inactivation of *p16* may cause abnormal cell cycles and uncontrolled cell growth (Huschtscha and Reddel, 1999). Recent data have indicated inactivation of *p16* in HCC is due to de novo hypermethylation of the 5′-promotor-associated CpG island (Liew et al, 1999). Introduction of *ras* mutations in vivo into cells deficient in *p16* (INK4a) is sufficient to induce characteristics of cellular transformation such as tumour formation (Gressani et al, 1998; Pantoja and Serrano, 1999; Rodrigues-Puebla et al, 1999). Data from K-*ras* transformation studies suggest that K-*ras* mutations induce *p16* hypermethylation and inactivation of *p16* (MacLeod et al, 1995; Serrano et al, 1995; Wadhwa et al, 2000).

To analyse the K-*ras*-2 oncogene and its possible association with *p16* methylation status, we examined tissue from 18 patients with resected HCCs and known VC exposure. All patients had received financial compensation after extensive assessment to

*Received 2 October 2000*
*Revised 18 December 2000*
*Accepted 18 December 2000*

*Correspondence to:* A Tannapfel

**Table 1** Patients data

| Case no | Age (y) | Exposure duration (mos) | Exposure amount (ppm – years) | Occupation | Histopathological diagnosis | K-*ras*-2 status | | p16 status |
|---|---|---|---|---|---|---|---|---|
| | | | | | | Carcinoma | Non-neoplastic liver tissue | |
| 1 | 56 | 233 | 7900 | autoclave cleaner | well diff. HCC without cirrhosis | wild type | wild type | methylated |
| 2 | 64 | 241 | 9700 | autoclave cleaner | mod. diff. HCC in cirrhosis | wild type | wild type | wt |
| 3 | 59 | 250 | 9700 | autoclave operator | mod. diff. HCC in cirrhosis | mutant | wild type | methylated |
| 4 | 62 | 159 | 4200 | mechanic/maintenance worker | mod.diff. HCC without cirrhosis | wild type | wild type | LOH[a] |
| 5 | 53 | 224 | 3600 | autoclave cleaner | well diff. HCC without cirrhosis | mutant | mutant | methylated |
| 6 | 66 | 230 | 5600 | autoclave cleaner | poorly diff. HCC without cirrhosis | wild type | wild type | methylated |
| 7 | 51 | 301 | 11700 | autoclave cleaner | well diff. HCC without cirrhosis | wild type | wild type | methylated |
| 8 | 74 | 399 | 12700 | autoclave cleaner | mod.diff. HCC without cirrhosis | wild type | wild type | wt |
| 9 | 69 | 288 | 11700 | autoclave cleaner | well diff. HCC in cirrhosis | wild type | mutant | methylated |
| 10 | 75 | 304 | 12000 | autoclave cleaner | mod.diff. HCC without cirrhosis | mutant | wild type | wt |
| 11 | 56 | 240 | 10500 | autoclave cleaner | well diff. HCC without cirrhosis | mutant | mutant | methylated |
| 12 | 71 | 416 | 20000 | autoclave cleaner | mod.diff. HCC without cirrhosis | mutant | wild type | methylated |
| 13 | 64 | 184 | 7000 | autoclave worker | mod.diff. HCC without cirrhosis | wild type | wild type | methylated |
| 14 | 49 | 181 | 7500 | autoclave cleaner | poorly diff. HCC without cirrhosis | wild type | wild type | methylated |
| 15 | 58 | 124 | 3700 | mechanic/maintenance worker | mod.diff. HCC without cirrhosis | wild type | wild type | wt |
| 16 | 64 | 262 | 8700 | autoclave operator | well. diff. HCC without cirrhosis | wild type | wild type | methylated |
| 17 | 68 | 171 | 5200 | autoclave cleaner | mod. diff. HCC without cirrhosis | wild type | wild type | methylated |
| 18 | 57 | 205 | 8500 | autoclave cleaner | mod.diff. HCC without cirrhosis | mutant | wild type | methylated |

[a] LOH: with the two markers D9S 1751 and D9S171. wt = wild type.

© 2001 Cancer Research Campaign prove the association of their cancer with VC exposure. As a control group, hepatocellular carcinoma tissue from 20 patients with chronic hepatitis B and C virus infection and alcohol-induced cirrhosis with HCC was used. The presence of K-*ras*-2 mutations was examined by direct sequencing after microdissection of the tumour nodules.

### MATERIALS AND METHODS

### Patients and tissue samples

Data were extracted from the German industrial professional association for the chemical industry which is responsible for statutory accident insurance and provides medical and social rehabilitation and financial compensation to persons who incur an industrial injury or occupational disease. According to the German social legislation code VII, compensation for work-related impairment and disability is only possible in cases of a proven association of occupational factor and disease. The criteria for the patients included in this study were a documented chronic quantitated exposure to VC, which leads to compensation under the German social legislation code VII (Table 1). Estimates of VC exposure were based on years worked with VC and estimated ppm years of VC exposure. Based on the exposure matrix of Heldaas et al (1984) for production, autoclave cleaning, maintenance and packing/drying job categories, exposures averaged 2000 ppm from 1950 through 1954, 1000 ppm from 1955 through 1959, 500 ppm from 1960 through 1967, 100 ppm from 1968 through 1974 and 1 ppm thereafter. 18 patients diagnosed with HCC who had undergone partial hepatectomy between 1981 and 1997 were identified and included all males with an average age of 62 years (49 to 75 y) and an average exposure to VC of 245 months (124 to 416 months). The average amount of exposure to VC was 8883 ppm (range 3600–20 000 ppm) and occurred between 1935 to 1984. The latency period between first exposure and the initial histological diagnosis of HCC varied between 10 and 25 years with a median latency of 18 years.

All other aetiologies of HCC were excluded by clinical history, laboratory serological examination and immunohistochemical analysis.

As a control group, 20 patients with hepatocellular carcinoma – most certainly without VC exposure – were analysed. These hepatocellular carcinomas had a known, clinically and serologically defined aetiology. They were attributed to chronic hepatitis B ($n$ = 7) and hepatitis C virus infection ($n$ = 5). 8 patients with liver cirrhosis after long-term alcohol consumption and HCC were also included in the control group (Table 2).

Formalin-fixed blocks of tissue with tumour and adjacent non-neoplastic liver tissue was available for analysis. All tumours were diagnosed as HCCs. Each tumour was re-evaluated with regard to typing, staging and grading (WHO, 1994; UICC, 1997). Tumour typing and staging was performed using WHO and UICC (1997) criteria.

### DNA samples

For each HCC sample, the lesions were first identified on routinely stained haematoxolin and eosin slides (H&E). Additional sections were cut from the paraffin blocks with a microtome set at 6 μm, and dried on slides overnight at 37˚C. Tumour and adjacent non-neoplastic tissue was identified and microdissected after rapid staining with H&E. The tissue was removed from the slide under solution (25 μl Tris buffer, 0.05 mol) with the tip of a sealed glass pipette and then evacuated into a microcapillary. The samples were transferred to Eppendorf tubes and incubated with proteinase K at 37˚C overnight. Proteinase K activity was inactivated by heating to 95˚C for 10 min, and the resulting solutions were used directly as templates for K-*ras* analyses. Following proteinase K incubation, the DNA was extracted in standard fashion, twice in

**Table 2** Patients data of the control group

| Case no. | Age (y) | UICC – Stage | Aetiology | Histopathological diagnosis | K-*ras*-2 status | | p16 status |
|---|---|---|---|---|---|---|---|
| | | | | | Carcinoma | Non-neoplastic liver tissue | |
| 1 | 67 | IIIA | Hepatitis B | well diff. HCC without cirrhosis | wild type | wild type | methylated |
| 2 | 78 | II | Hepatitis B | mod.diff. HCC in cirrhosis | wild type | wild type | wt |
| 3 | 49 | I | Hepatitis B | mod. diff. HCC without cirrhosis | mutant | wild type | wt |
| 4 | 76 | IIIB | Hepatitis B | mod.diff. HCC without cirrhosis | wild type | wild type | wt |
| 5 | 57 | IVA | Hepatitis B | well diff. HCC in cirrhosis | mutant | mutant | methylated |
| 6 | 34 | II | Hepatitis B | poorly diff. HCC without cirrhosis | wild type | wild type | methylated |
| 7 | 59 | IIIA | Hepatitis B | well diff. HCC without cirrhosis | wild type | wild type | methylated |
| 8 | 54 | IIIB | Hepatitis C | mod.diff. HCC without cirrhosis | wild type | wild type | LOH |
| 9 | 63 | II | Hepatitis C | well diff. HCC in cirrhosis | wild type | wild type | methylated |
| 10 | 71 | IIIB | Hepatitis C | mod.diff. HCC in cirrhosis | mutant | wild type | wt |
| 11 | 58 | IVA | Hepatitis C | well diff. HCC without cirrhosis | wild type | wild type | methylated |
| 12 | 80 | IIIB | Hepatitis C | mod.diff. HCC without cirrhosis | wild type | wild type | methylated |
| 13 | 55 | II | Alcohol consumption | mod.diff. HCC in cirrhosis | wild type | wild type | methylated |
| 14 | 51 | I | Alcohol consumption | poorly diff. HCC in cirrhosis | wild type | wild type | methylated |
| 15 | 65 | IIIA | Alcohol consumption | mod.diff. HCC without cirrhosis | wild type | wild type | wt |
| 16 | 62 | IIIA | Alcohol consumption | well. diff. HCC in cirrhosis | wild type | wild type | methylated |
| 17 | 72 | II | Alcohol consumption | mod.diff. HCC in cirrhosis | wild type | wild type | wt |
| 18 | 75 | IIIB | Alcohol consumption | well diff. HCC in cirrhosis | wild type | wild type | wt |
| 19 | 61 | IVA | Alcohol consumption | well diff. HCC in cirrhosis | wild type | wild type | methylated |
| 20 | 52 | IIIB | Alcohol consumption | mod.diff. HCC in cirrhosis | wild type | wild type | LOH |

[a]LOH: with the two markers D9S 1751 and D9S171. wt = wild type.

*© 2001 Cancer Research Campaign*

phenol and twice in chloroform, then followed by ethanol precipitation.

### K-*ras*-2 mutation analysis

All pre-PCR tissue was handled in an environment free of PCR products. All samples were coded and the investigator was blinded to all patients' clinical details. De-paraffinized tissue was recovered with 2 cycles each of a 15 min incubation with xylene followed by centrifugation for 5 minutes at 14 000 rpm. The tissue pellet was washed twice in absolute ethanol and twice in phosphate buffered saline. The pellet was incubated with 10 pellet volumes (approximately 500 µl) of lysis buffer (0.32 M sucrose, 10 mM Tris-HCl, 1% (v/v) Triton X-100) and 0.2 volumes of proteinase K (final concentration 400 µg $ml^{-1}$) for 2 to 3 days at 37˚C. DNA was phenol-chloroform extracted and precipitated in ethanol using conventional techniques (Bjorheim et al, 1998). The resulting DNA pellet was resuspended in 50 µl TE buffer, pH 7.4, (10 mM Tris-HCl, pH 7.4, 1 mM EDTA, pH 8.0). DNA samples were stored at 20˚C.

The first exon of K-*ras*-2 was amplified by PCR using primers designed to avoid amplification of the K-*ras* pseudogene (Bautista et al, 1997; Bjorheim et al, 1998). The primers were 5′-ATTATAAGGCCTGCTGAAAATG-ACTGA-3′ (upstream primer) and 5′-ATATGCATATTAAAACAAGATTTACCT-CTA-3′ (downstream primer) which yield a 155 base-pair product. Amplification was performed using a PCR technique (Bjorheim et al, 1998) from 63˚C to 53˚C over 10 cycles, followed by 30 cycles at 94˚C, 53˚C and 72˚C. PCR products were purified using the Qiaquick PCR purification kit (Qiagen, Hilden, Germany) and sequenced using dye primer cycle sequencing and AmpliTaq polymerase FS on an Applied Biosystems 373 DNA sequencer (ABI 373; Applied Biosystems-Perkin-Elmer/Cetus, Norwalk).

### Controls

DNA from colon carcinoma cell lines SW480 (Clontech, Palo Alto, California, USA) and HCT116 (American Type Culture Collection, ATCC, Rockville, Maryland, USA) with known K-*ras*-2 mutations at codon 12 (GTT) and codon 13 (GAC) respectively, were used as positive controls in each of the parallel procedures. Negative controls, without DNA, were run as controls for contamination.

If a mutation was detected, confirmation by amplification and sequencing of a fresh DNA sample using the upstream primer was performed. Any sequences which proved difficult to read were re-amplified and re-sequenced.

### Methylation specific PCR (MSP)

The CpG WIZ *p16* Methylation Assay Kit was used (OncorInc, Gaithersburg, MD, USA) according to the manufacturers instructions. After an initial bisulphide reaction to modify the DNA, a PCR amplification with specific primers was performed to distinguish methylated from unmethylated DNA. Primers were specific for the unmethylated *p16* (5- TTATTAGAGGGTGGGGT-GGATTGT-3, 5-CAACCCCAAACCACAACCATAA-3) or the methylated *p16* (5-TTATTAGAGGGTGGGGCGGATCGC-3, 5-GACCCCGAACCGCGACCGTAA-3). A sample of 7 µg 100 $\mu l^{-1}$ DNA was denaturated by NaOH, 0.2M, for 10 min at room temperature. DNA Modification Reagent I was added, incubated for 24 h at 50˚C and subsequently purified by DNA Modification Reagent II and III in the presence of 50 µl water. The bisulphate modification of DNA was completed with 0.3M NaOH treatment for 5 min, followed by ethanol precipitation. For hot start PCR, the PCR mixture contained Universal PCR Buffers (1X9, 4dNTP's (1.25 nM), U or M primers (300 ng each per reaction)). Annealing temperature was 65˚C for 35 cycles. The PCR product was directly electrophoresed on a 3% agarose gel, stained with ethidium bromide and visualized under UV illumination. Bisulphite-converted DNA from adjacent non-neoplastic liver tissue from each patient served as a negative control indicated by the presence of the unmethylated but not the methylated band.

### Microsatellite analysis of the loss of heterozygosity (LOH)

We utilized 9 microsatellite markers flanking the region of chromosome 9p21 region where *p16* is located. They were D9S161, D9S126, D9S171, D9S1752, D9S1748, D9S1747, D9S1749, D9S1751, and IFNA obtained from Research Genetics (Huntsville AL, USA). The primers were labelled with $^{32}$P-ATP. PCR amplification was performed in a 10 µl reaction volume including 30 ng genomic DNA, 10 mM TrisHCl, 50 mM KCl, 1.5 mM $MgCl_2$, 62.5 µM deoxynucleotide triphosphate, 0.1 U Taq DNA-polymerase and 1 pmol of each primer. A total of 45 cycles was performed with annealing temperatures of 53˚ and 55˚C. PCR products were subsequently electrophoresed, dried and autoradiographed. Homozygous deletion of the *p16* gene was confirmed by comparative PCR involving the amplification of 2 different sets of primer pairs in the same reaction mixture. For homozygous deletions, the control marker D9S126 was used. Homozygous deletion was scored if the signal intensity (assessed by visual examination and densitometer) of the *p16* in tumour tissues was at least 10-fold less than the signal from the non-neoplastic tissues. The intensity of the D9S126 control allele was approximately equal in tumour and adjacent non-neoplastic liver.

### DNA-sequencing of the p16 gene

For mutation analysis on exon 1, 2 (2A, 2B, and 2C) and 3, SSCP analysis was employed using the primers described by Hussussian et al (1994). The SSCP analysis was performed as previously described (Tannapfel et al, 1999a). The primers were labelled with $^{32}$P-ATP and each sample underwent PCR analysis (denaturing for 30 s, annealing for 45 s, extension for 30 s at 94˚C, 55–60˚C and 72˚C respectively). The PCR products were electrophoresed, the gels dried and autoradiographed. Variant SSCP bands were cut out from the gel and the DNA eluted. Variants bands and 3 µl of the eluted DNA were used as template for unlabelled PCR. After purification of the PCR products, sequencing analysis was performed using the DNA-Sequenase-Kit (Amersham, Germany) and an automatic sequencing analyser (ABI 373; Applied Biosystems-Perkin-Elmer, Germany). All mutations found were confirmed by direct sequencing of the amplified tumour and adjacent non-neoplastic DNA to identify germline mutations and polymorphisms. The sequences of all primers used for amplification are available from the authors upon request.

© 2001 Cancer Research Campaign 

**Table 3** K-*ras*-2 mutations and p16 methylation status in VC-associated hepatocellular carcinoma

| Case | Stage | Grade | Codon | Mutation substitution | Amino acid | p21ras IHC[a] | p16 methylation[b] |
|---|---|---|---|---|---|---|---|
| Codon 12 | | | | GGT (wild type) | glycine | | |
| 3 | IVA | 2 | 12 | GGT→GAT | aspartate | ++ | ++ |
| 5 | IIIA | 1 | 12 | GGT→GTT | valine | ++ | ++ |
| 10 | IIIB | 2 | 12 | GGT→TGT | cystine | ++ | – |
| 12 | IIIB | 2 | 12 | GGT→GAT | aspartate | + | ++ |
| Codon 13 | | | | GGC (wild type) | glycine | | |
| 11 | II | 1 | 13 | GGC→TGC | cystine | ++ | ++ |
| 18 | IVA | 2 | 13 | GGC→GAT | aspartate | + | ++ |
| 5 | non-neoplastic tissue | | 12 | GGT→GAT | aspartate | + | – |
| 9 | non-neoplastic (cirrhotic) tissue | | 13 | GGC→CAT | aspartate | + | – |
| 11 | non-neoplastic tissue | | 13 | GGC→GAC | aspartate | ++ | – |

[a] ras immunohistochemistry (IHC): positivity assessment according to the literature (Thor et al, 1986): negative (–) <1% positive nuclei; weakly positive (+): single positive cells (10–30%); moderately positive (++): numerous positive cells (30–60%); strongly positive (+++): more than 60% positive tumour cells.
[b] p16 methylation status: ++ methylation detection; — not detected.

### Immunohistochemical analysis

The immunohistochemical analysis was performed as previously described (Tannapfel et al, 1999b). In all cases, tumour and non-neoplastic liver tissue was examined. The following antibodies were used: ras (ras, monoclonal Clone F132; mouse, dilution: 1:120; final concentration: 10 μg $ml^{-1}$, Boehringer[R] Mannheim, Germany), *p16* (polyclonal; rabbit, dilution 1:500, Pharmingen[R], San Diego, CA), AFP (polyclonal; rabbit, dilution 1:300, Dianova[R], Hamburg, GERMANY), Hepatitis B (HBs-Ag, dilution: 1:80, Dako-Diagnostik[R], Hamburg, Germany), and Hepatitis C (HCV, Clone TORDJ1-22, dilution: 1:200, BioGenex[R], Hamburg, Germany). Positive controls of antigen-containing tissue were included in each batch. Negative controls, which had the primary antibody replaced by mouse or goat ascites fluid, were also included (Sigma-Aldrich Biochemicals, St Louis, MO).

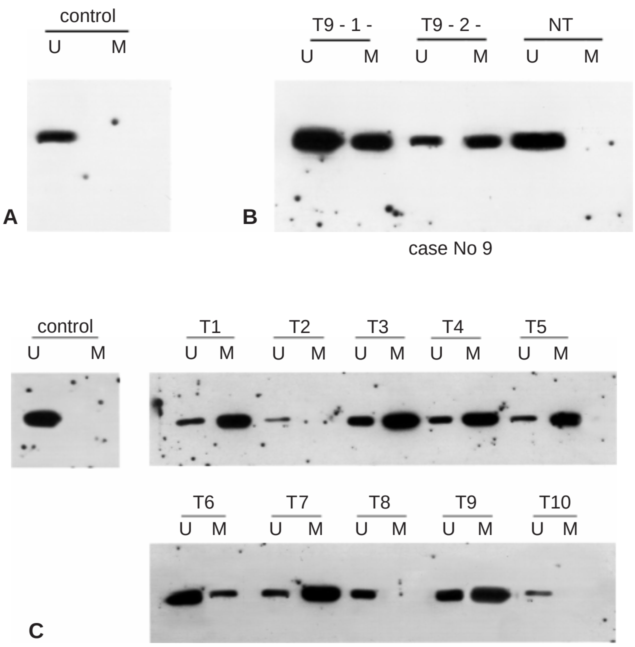

**Figure 1** Methylation analysis of p16 in hepatocellular carcinomas associated with VC. MSP results are expressed as unmethylated p16-specific bands (U) or methylated p16-specific bands (M). (**A**) Bisulfite-converted DNA from normal liver tissue (N) served as a negative control as indicated by the presence of the U but not the M band. (**B**) MSP results of case No. 9. Two tumour-nodules (T9–1- and T9–2-) with methylated p16. The tumour surrounding non-neoplastic liver tissue (NT) with unmethylated p16. (**C**) Representative cases of VC-associated hepatocellular carcinoma. The numbers of the hepatocellular carcinomas are shown above the corresponding lanes and are identical to Tables 1 and 2

### RESULTS

### K-*ras*-2 status

PCR amplification and DNA sequencing enabled detection of heterozygous mutations in 6 of 18 VC-HCCs (33%). 4 patients (22%) had a mutation of codon 12 and 2 of codon 13. None showed multiple mutations. In 3 cases (17%), mutation of the K-*ras*-2 gene was detected in adjacent non-neoplastic liver tissue; 2 mutations occurred in codon 13 and one was found in codon 12. In patient No 9 (Table 3), a mutation occurred in cirrhotic liver tissue, while the tumour exhibited a wild type K-*ras*-2. The base pair change consisted of a GGC → CAT mutation which lead to a substitution of glycine for aspartatic acid. We failed to observe an association between K-*ras*-2 mutation pattern and duration or amount of VC exposure.

Within the 20 HCCs of the countrol group, specific K-*ras*-2 mutation were found in 3 cases (15%). 2 cases were attributed to chronic hepatitis B virus infection (GGT → GTT and GGC → GAC). In one case, the hepatocellular carcinoma arrised within a hepatitis C virus-infected liver (GGT → TGT). K-*ras*-2 mutations were also detected in liver cirrhosis attributed to hepatitis B virus infection (GGC → TGC) (Table 2).

Performing a semiquantitative assessment of $p21^{ras}$ immunoreactivity, neither the staining intensity nor the number of positive cells correlated to the mutation pattern or to other histo-pathological parameters (Table 3). Non-neoplastic liver tissue was occasionally positive for $p21^{ras}$.

### p16 alterations

The methylation-specific PCR demonstrated aberrant methylation at the 5′ CpG island of the *p16* gene in 13 of 18 (72%) VC-associated carcinomas (Table 3, Figure 1). Despite microdissection, amplification of unmethylated templates were detected most likely due to contaminating normal intratumorous tissue (fibroblasts, endothelial

 © 2001 Cancer Research Campaign

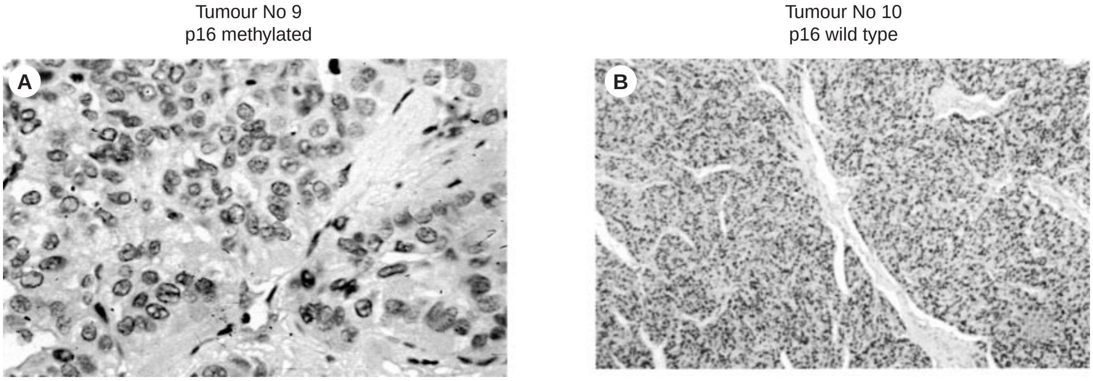

**Figure 2** Immunostaining of p16 protein in hepatocellular carcinoma. (**A**) Patient No 9 (same patients as in Figure 1 B) with a methylated p16 gene. Complete loss of p16 expression. (**B**) Immunohistochemical staining of p16 in a moderately differentiated hepatocellular carcinoma without p16 methylation. Strong nuclear positivity of the tumour cells (brown reaction product). (Original magnification × 40)

cells, inflammatory cells). In corresponding non-neoplastic liver tissue, methylated templates were amplified in one case. This patient exhibited a cirrhosis with a high degree of inflammation. Of 6 carcinomas with K-*ras*-2 mutations, 5 specimens also showed methylated *p16* (Table 3). All 13 cases with aberrant methylation of the *p16* gene showed a complete loss of immunoreactivity to p16 protein within the tumour tissue (Figure 2A). In 4 cases without a methylated *p16* promotor defined by methylation-specific PCR, a nuclear staining of *p16* protein was observed in nearly all tumour cells with a moderate to high intensity of immunoreactivity (Figure 2B). In normal liver tissue, *p16* protein was detected in all cases. One HCC showed LOH in at least one locus on chromosome 9p21. The highest frequency of LOH was observed with the microsatellite marker D9S 1751 (3 cases) and the lowest with D9S171 (one case). Homozygous deletion of the *p16* gene was not observed. We failed to observe specific mutations of the *p16* gene. In SSCP analysis, a mobility shift was detected in one carcinoma, however, we did not find a specific mutation of *p16* in this tumour.

11 out of 20 hepatocellular carcinomas of the control group (55%) exhibited a methylated p16 promotor (Table 2). Immunohistochemistry revealed a p16 protein loss of these cases. 2 HCC of this group showed LOH in at least one marker. We failed to identify homozygous deletion of the p16 locus.

### Histopathological features

The pathological data (stage and grade) of VC-associated HCC are summarized in Tables 1 and 3. Within the adjacent non-neoplastic liver tissue, histopathological changes associated with VC exposure were observed to a variable degree. In the 15 non-cirrhotic cases, a moderate, predominantly microvesicular steatosis was present with homogeneous distribution within the liver acinus. In 8 of these cases, an interstitial and perivenular fibrosis was observed. Generally, a mild non-specific mesenchymal inflammatory reaction occurred with an increased number of Kupffer cells and prominent sinus endothelial cells. In 5 cases, a marked sinusoidal dilatation was observed resembling foci of peliosis. In one case we observed a local portal phlebitis. We failed to observe a significant correlation between the extent of histopathological liver damage and duration of exposure.

Immunohistochemical analysis revealed AFP immunoreactivity in 12 of 18 HCC (67%). All cases were negative for HBs-, or HBc- or HCV-antigen expression within the non-neoplastic liver tissue as well as within the tumour.

### DISCUSSION

Liver angiosarcomas (LAS) were the first proven primary liver tumours encountered in workers exposed to VC, while to date only few HCCs in workers exposed to VC have been reported (Saurin et al, 1997). Saurin et al have reported 2 histologically proven cases of HCC after VC exposure. A recently published cohort study on workers with chronic exposure to VC reported a significant increase in primary liver cancer (Du and Wang, 1998). In that data, the risk of developing a HCC increased with the total cumulative exposure and the time since first exposure. Published studies have to date not excluded the classic risk factors for HCCs. Based on data recruited from the German industrial professional association for the chemical industry, we examined 18 patients with documented chronic quantitated exposure to VC and who carried the diagnosis of HCC. All patients had received financial compensation for their work-related impairment due to the proven relationship between work (VC exposure) and occurrence of disease (primary tumour of the liver). All 18 patients lacked other discernable risk factors for developing liver cirrhosis and/or HCC. In our series, an important argument for an association of VC and HCC is the absence of other known risk factors in the examined patients. Hepatitis B- or C-virus infection were ruled out serologically and also immunohisto-chemically. Drugs, metabolic disorders or autoimmune chronic hepatitis were excluded by laboratory investigation and careful clinical history. Ethanol abuse could not be completely evaluated in these patients; however, the absence of histological features of heavy chronic alcoholism (Mallory bodies, pericellular fibrosis, severe steatosis, alcoholic hepatitis) argue against a significant impact of ethanol consumption in the aetiology of HCC in these patients. The 3 patients with concomitant liver cirrhosis exhibited no classical features of alcohol-induced cirrhosis. Mild to moderate alcohol intake may be a potential cocarcinogenic factor in VC-associated HCC but we present no evidence for or against that argument. In Western countries, only 10–20% of all HCCs arise in non-cirrhotic livers (Tannapfel et al, 1999c). In contrast, only 3 of our patients had a concomitant liver cirrhosis, a fact further supporting VC as the likely carcinogen and again ruling out the classical risk factors for the development of HCCs.

In our series, the average duration of exposure and the amount of exposure was 245 months and 8880 ppm years, respectively, which corresponds with the current literature (Marion et al, 1996).

© 2001 Cancer Research Campaign

The observed mean latency period between first VC exposure and the initial diagnosis of HCC was 18 years (range 10 to 25 years) which is significantly longer than the observed latency for VC-associated LAS (Hollstein et al, 1994). One may speculate that VC has a lesser carcinogenic effect on hepatocytes compared to endothelial cells or that endothelial cells may have defects in their DNA repair enzyme (Barbin et al, 1997; Marion, 1998). In contrast to VC-induced LAS, which has a specific mutation pattern of the K-*ras*-2 oncogene as described by Marion (1998), there remains a lack of data concerning mutations in VC-associated HCC. We therefore performed sequencing analysis of the K-*ras*-2 gene in order to investigate a possible link between carcinogen exposure and cancer. In our study, we found a high prevalence of G → A point mutations within codon 12 and 13. All 3 K-*ras*-2 mutations occurring in non-neoplastic liver tissue were G → A point mutations as well. In contrast, G → A mutations were found in one case of the control group. This supports the data from *ras* mutations studies in LAS of VC-exposed workers, where 83% of the tumours were found to contain G → A point mutations at codon 13. Exposure to VC produced a similar mutation pattern with G:C → A:T point mutations at codon 61 of the N-*ras* gene in liver tumours in Sprague-Dawley rats (Froment et al, 1994; Boivin-Angele, 2000). Collectively, these observations suggest that VC induces a high frequency G:C → A:T point mutations. The carcinogenic metabolite of VC, chloroethylene oxide (Przygodzki et al, 1997; Roy et al, 1998; Barbin, 1999) and a major promutagenic DNA adduct of vinyl chloride, N2,3-ethenoguanine (Dogliotti et al, 1998), produce these same mutations.

*Ras* oncogene mutations are frequently found in chemically induced HCC in experimental animals (Chao et al, 1999; Boivin-Angele et al, 2000; Xia et al, 1998), in contrast the frequency of *ras* mutations in human HCC is much less. To date, there are few studies on the prevalence of *ras* mutations in sporadic HCC of various aetiologies. In a series of 19 sporadic HCCs, mutations of the N-*ras* gene were found in about 16% (Challen et al, 1992). However, the authors analysed the *ras* mutation status using PCR-based mutation-specific oliginucleotide analyses and did not perform DNA sequencing which may led to an underestimation of the mutation rate. We utilized microdissection of our lesions followed by very sensitive PCR methods which detect one mutated K-*ras* codon 12 or 13 allele in the presence of 10 000–100 000 copies of the wild-type allele (Norheim Andersen et al, 1996). In our control series of HCC in patients without VC exposure, we could detect specific K-*ras*-2 mutations in about 15%. Further studies of sporadic HCC are currently in progress to search for the prevalence of K-*ras*-2 mutations in a large number of patients.

Our data show a high frequency of *p16* hypermethylation in HCC which supports recent data published by the literature (Matsuda et al, 1999). De novo hypermethylation of *p16* is reported to be the most common mechanism for inactivation of this cell-cycle inhibitor (Matsuda et al, 1999). Nearly all carcinomas harbouring K-*ras*-2 mutations subsequently contained *p16* methylation in our data. Overexpression of the *p16* gene blocks S-phase progression of the cell cycle and inhibits *ras*-induced cell proliferation (Serrano et al, 1997). Oncogenic *ras* can transform most immortal rodent cells to a tumourigenic state (Serrano et al, 1995). In contrast, expression of oncogenic *ras* in primary human or rodent cells results in a permanent $G_1$ arrest which is accompanied by accumulation of p53 or p16 (Pantoja and Serrano, 1999). However, transformation of primary cells by *ras* requires either a cooperating oncogene or the inactivation of tumour suppressors such as *p53* or *p16* (Serrano et al, 1997). One may speculate, that inactivation of *p16* releases cells from *ras*-induced $G_1$ arrest and allow them to enter the cell cycle. These data imply that aberrant expression of *p16* and *ras* mutation represent fundamental defects in cell cycle regulation and together may play an important role in the pathogenesis of HCC.

In summary, our study is the first to characterize the K-*ras*-2 gene mutation in VC-associated HCCs. The mutation spectrum we found with a high prevalence of G → A point mutations even in adjacent non-neoplastic liver tissue of patients exposed to VC supports those reported for the carcinogenic metabolites of VC. Our findings of a strong correlation between *p16* methylation and the K-*ras*-2 mutation in a given tumour could indicate a close molecular link between K-*ras*-2 and *p16* genes in HCCs.

### ACKNOWLEDGEMENTS

We would like to express our gratitude to Dr jur B Koch, director of the German Industrial Professional Association for the Chemical Industry, Cologne (Germany), for the excellent support in collecting the patients data. The authors thank Mr Danny Milner for excellent assistance in preparing the manuscript.

This work was supported by the Johannes and Frieda-Marohn-Stiftung, Erlangen, Germany.

### REFERENCES

Barbin A (1999) Role of etheno DNA adducts in carcinogenesis induced by vinyl chloride in rats. *IARC Sci Publ* **150**: 303–313

Barbin A, Froment O, Boivin S, Marion MJ, Belpoggi F, Maltoni C and Montesano R (1997) p53 gene mutation pattern in rat liver tumors induced by vinyl chloride. *Cancer Res* **57**: 1695–1698

Bautista D, Obrador A, Moreno V, Cabeza E, Canet R, Benito E, Bosch X and Costa J (1997) Ki-ras mutation modifies the protective effect of dietary monounsaturated fat and calcium on sporadic colorectal cancer. *Cancer Epidemiol Biomarkers Pre* **6**: 57–61

Begley TJ, Haas BJ, Noel J, Shekhtman A, Williams WA and Cunningham RP (1999) A new member of the endonuclease III family of DNA repair enzymes that removes methylated purines from DNA. *Curr Biol* **17**: 653–656

Bjorheim J, Lystad S, Lindblom A, Kressner U, Westring S, Wahlberg S, Lindmark G, Gaudernack G, Ekstrom P, Roe J, Thilly WG and Borresen-Dale A (1998) Mutation analyses of KRAS exon 1 comparing three different techniques: temporal temperature gradient electrophoresis, constant denaturant capillary electrophoresis and allele specific polymerase chain reaction. *Mutat Res* **403**: 103–112

Boivin-Angele S, Lefrancois L, Froment O, Spiethoff A, Bogdanffy MS, Wegener K, Wesch H, Barbin A, Bancel B, Trepo C, Bartsch H, Swenberg J and Marion MJ (2000) Ras gene mutations in vinyl chloride-induced liver tumours are carcinogen-specific but vary with cell type and species. *Int J Cancer* **85**: 223–2273

Challen C, Guo K, Collier JD, Cavanagh D and Bassendine MF (1992) Infrequent point mutations in codons 12 and 61 of ras oncogenes in human hepatocellular carcinomas. *J Hepatol* **14**: 342–346

Chao HK, Tsai TF, Lin CS and Su TS (1999) Evidence that mutational activation of the ras genes may not be involved in aflatoxin B(1)-induced human hepatocarcinogenesis, based on sequence analysis of the ras and p53 genes. *Mol Carcinog* **26**: 69–73

DeVivo I, Marion MJ, Smith SJ, Carney WP and Brandt-Rauf PW (1994) Mutant c-Ki-ras p21 protein in chemical carcinogenesis in humans exposed to vinyl chloride. *Cancer Causes Control* **5**: 273–278

Dogliotti E, Hainaut P, Hernandez T, D'Errico M and DeMarini DM (1998) Mutation spectra resulting from carcinogenic exposure: from model systems to cancer-related genes. *Rec Res Cancer Res* **154**: 97–124

Du CL and Wang JD (1998) Increased morbidity odds ratio of primary liver cancer and cirrhosis of the liver among vinyl chloride monomer workers. *Occup Environ Med* **55**: 528–532

© 2001 Cancer Research Campaign el Ghissassi F, Barbin A and Bartsch H (1998) Metabolic activation of vinyl chloride by rat liver microsomes: low-dose kinetics and involvement of cytochrome P450 2E1. *Biochem Pharmacol* **55**: 1445–1452
Evans DMD, Jones WW and Kung ITM (1983) Angiosarcoma and hepatocellular carcinoma in vinyl chloride workers. *Histopathology* **7**: 377–388
Froment O, Boivin S, Barbin A, Bancel B, Trepo C and Marion MJ (1994) Mutagenesis of ras protooncogenes in rat liver tumors induced by vinyl chloride. *Cancer Res* **54**: 5340–5345
Gressani KM, Rollins LA, Leone-Kabler S, Cline JM and Miller MS (1998) Induction of mutations in Ki-ras and INK4a in liver tumors of mice exposed in utero to 3-methylcholanthrene. *Carcinogenesis* **19**: 1045–1052
Heldaas SS, Langard SL and Andersen A (1984) Incidence of cancer among vinyl chloride workers. *Br J Industr Med* **41**: 25–30
Hollstein M, Marion MJ, Lehmann T, Welsh J, Harris CC, Martel-Planche K, Kusters I and Montesano R (1994) p53 mutations at A:T base pairs in angiosarcomas of vinyl chloride-exposed factory workers. *Carcinogenesis* **15**: 1–3
Huschtscha LI and Reddel RR (1999) p16(INK4a) and the control of cellular proliferative life span. *Carcinogenesis* **20**: 921–926
Hussussian CJ, Struewing JP, Goldstein AM, Higgins PA, Ally DS, Sheahan MD, Clark WH Jr, Tucker MA and Dracopoli NC (1994) Germline p16 mutations in familial melanoma. *Nat Genet* **8**: 15–21
Liew CT, Li HM, Lo KW, Leow CK, Chan JY, Hin LY, Lau WY, Lai PB, Lim BK, Huang J, Leung WT, Wu S and Lee JC (1999) High frequency of p16INK4A gene alterations in hepatocellular carcinoma. *Oncogene* **18**: 789–795
Luo JC, Liu HT, Cheng TJ, Du CL and Wang JD (1998) Plasma Asp13-Ki-ras oncoprotein expression in vinyl chloride monomer workers in Taiwan. *J Occup Environ Med* **40**: 1053–1058
MacLeod AR, Rouleau J and Szyf M (1995) Regulation of DNA methylation by the Ras signaling pathway. *J Biol Chem* **270**: 11327–11337
Marion MJ (1998) Critical genes as early warning signs: example of vinyl chloride. *Toxicol Lett* 102–103: 603–607
Marion MJ, DeVivo I, Smith S, Luo JC and Brandt-Rauf PW (1996) The molecular epidemiology of occupational carcinogenesis in vinyl chloride exposed workers. *Int Arch Occup Environ Health* **68**: 394–398
Matsuda Y, Ichida T, Matsuzawa J, Sugimura K and Asakura H (1999) p16(INK4) is inactivated by extensive CpG methylation in human hepatocellular carcinoma. *Gastroenterology* **116**: 394–400
Norheim Andersen S, Breivik J, Lovig T, Meling GI, Gaudernack G, Clausen OP, Schjolberg A, Fausa O, Langmark F, Lund E and Rognum TO (1996) K-ras mutations and HLA-DR expression in large bowel adenomas. *Br J Cancer* **74**: 99–108
Pantoja C and Serrano M (1999) Murine fibroblasts lacking p21 undergo senescence and are resistant to transformation by oncogenic Ras. *Oncogene* **18**: 4974–4982
Przygodzki RM, Finkelstein SD, Keohavong P, Zhu D, Bakker A, Swalsky PA, Soini Y, Ishak KG and Bennett WP (1997) Sporadic and Thorotrast-induced angiosarcomas of the liver manifest frequent and multiple point mutations in K-ras-2. *Lab Invest* **76**: 153–159
Rodriguez-Puebla ML, Robles AI and Conti CJ (1999) ras activity and cyclin D1 expression: an essential mechanism of mouse skin tumor development. *Mol Carcinog* **24**: 1–6
Roussel MF (1999) The INK4 family of cell cycle inhibitors in cancer. *Oncogene* **18**: 5311–5317
Roy R, Biswas T, Hazra TK, Roy G, Grabowski DT, Izumi T, Srinivasan G and Mitra S (1998) Specific interaction of wild-type and truncated mouse N-methylpurine-DNA glycosylase with ethenoadenine-containing DNA. *Biochemistry* **37**: 580–589
Saurin JC, Taniere P, Mion F, Jacob P, Üartensky C, Paliard P and Berger F (1997) Primary hepatocellular carcinoma in workers exposed to vinyl chloride. *Cancer* **79**: 1671–1677
Serrano M, Lin AW, McCurrach ME, Beach D and Lowe SW (1997) Oncogenic ras provokes premature cell senescence associated with accumulation of p53 and p16INK4a. *Cell* **88**: 593–602
Serrano M, Gomez-Lahoz E, DePinho RA, Beach D and Bar-Sagi D (1995) Inhibition of ras-induced proliferation and cellular transformation by p16INK4. *Science* **267**: 249–252
Soman NR and Wogan GN (1993) Activation of the c-Ki-ras oncogene in aflatoxin B1-induced hepatocellular carcinoma and adenoma in the rat: detection by denaturing gradient gel electrophoresis. *Proc Natl Acad Sci USA* **90**: 2045–2049
Tamburro Ch, Makk and L, Popper H (1984) Early hepatic histologic alterations among chemical (vinyl monomer) workers. *Hepatology* **4**: 413–418
Tannapfel A, Benicke M, Katalinic A, Uhlmann D, Kockerling F, Hauss J and Wittekind C (2000) Frequency of p16 (INK4A) alterations and K-ras mutations in intrahepatic cholangiocarcinoma of the liver. *Gut* **47**: 721–727
Tannapfel A, Geissler F, Köckerling F, Katalinic A, Hauss J and Wittekind Ch (1999b) Apoptosis and proliferation in relation to histopathological variables and prognosis in hepatocellular carcinoma. *J Pathol* **187**: 439–445
Tannapfel A, Wasner M, Krause K, Geissler F, Katalinic A, Hauss J, Mössner J, Engeland K and Wittekind Ch (1999c) Expression of p73 and ist relation to histopathology and prognosis in hepatocellular carcinoma. *J Natl Cancer Inst* **91**: 1154–1158
Thompson JA, Ho B and Mastovich SL (1985) Dynamic headspace analysis of volatile metabolites from the reductive dehalogenation of trichloro- and tetrachloroethanes by hepatic microsomes. *Anal Biochem* **142**: 376–384
Trivers GE, Cawley HL, DeBenedetti VMG, Hollstein M, Marion MJ, Bennett WP, Hoover ML, Prives CC, Tamburro CC and Harris CC (1995) Anti-p53 antibodies in sera of workers occupationally exposed to vinyl chloride. *J Natl Cancer Inst* **87**: 1400–1407
UICC: *TNM classification of malignant tumors* (1997) 5th ed. Sobin LH, Wittekind Ch, eds. New York: Wiley-Liss
Wadhwa R, Kaul SC and Mitsui Y (2000) Cellular mortality and immortalization: a complex interplay of multiple gene functions. *Prog Mol Subcell Biol* **24**: 191–204
WHO: *Histological typing of tumours of the liver* (1994) 2nd. Ishak T, ed. Berlin, Heidelberg, New York: Springer
Xia Q, Yi P, Zhan DJ, Von Tungeln LS, Hart RW, Heflich RH and Fu PP (1998) Liver tumors induced in B6C3F1 mice by 7-chlorobenz[a]anthracene and 7-bromobenz[a]anthracene contain K-ras protooncogene mutations. *Cancer Lett* **123**: 21–25
Xing EP, Nie Y, Wang LD, Yang GY and Yang CS (1999). Aberrant methylation of p16INK4a and deletion of p15INK4b are frequent events in human esophageal cancer in Linxian, China. *Carcinogenesis* **20**: 77–84

*© 2001 Cancer Research Campaign* 
